# Measuring associations between the food environment and dietary habits: comparing the proportion and density of food outlets

**DOI:** 10.1186/s12889-024-20976-x

**Published:** 2024-12-18

**Authors:** Kamille Almer Bernsdorf, Henrik Bøggild, Mette Aadahl, Ulla Toft

**Affiliations:** 1 Center for Clinical Research and Prevention, Copenhagen University Hospital - Bispebjerg and Frederiksberg, Copenhagen, Denmark; 2 Public Health and Epidemiology, Department of Health Science and Technology, Aalborg University, Aalborg, Denmark; 3 Department of Public Health, Section of Social Medicine, Faculty of Health and Medical Sciences, University of Copenhagen, Copenhagen, Denmark; 4 Steno Diabetes Center Copenhagen, Department for Prevention, Health Promotion and Community Care, Herlev, Denmark

**Keywords:** Geographic Information Systems, Built environment, Community food environment, Retail food environment, Dietary patterns

## Abstract

**Background:**

The food environment plays a crucial role in shaping our dietary choices and overall health. Spatial measures provide distinct perspectives on the physical food environment and its impact on diet. While proportion measures are theoretically considered to provide a more accurate representation of the overall physical food environment than density measures, it is important to recognize that the association between food environments and diet can vary depending on the context. Therefore, relying solely on one measure may not be appropriate.

**Methods:**

We systematically assessed the density and proportion of multiple food outlet types (fast-food outlets, convenience stores, supermarkets, and restaurants) around individuals homes using a large cross-sectional Danish study (*N* = 71,840). Densities were modeled in separate multilevel linear regression models, incorporating random intercepts from linear splines for each of the four food outlet types. Proportions were modeled without splines. Through the association with a dietary quality score (DQS), we examined the impact of quantifying the foodscape from density versus proportion measures. Associations were compared using parameter estimates, *p*-values, Akaike Information Criterion (AIC) values, and Akaike weights.

**Results:**

AIC values and Akaike weights were in favor of models including density measures. Across all outlet types, density measures were consistently negatively associated with the DQS until reaching densities of 3–5 (count/km2), at which point the direction of association became positive, indicating a shift towards a healthier DQS. After correcting for multiple comparisons, the most significant effect was observed for the sole significant proportion measure. A 10% increase in the proportion of fast-food outlets among "eating out options" was associated with a 7% decrease in the DQS, towards poorer dietary quality.

**Conclusions:**

The associations highlight that choosing food outlet density versus proportions to quantify the foodscape impact findings of substantial importance when considering the significance level and direction of association. Findings suggests a threshold effect when using density measures indicating abundance of many food outlets, at which the association with dietary quality alters significantly towards healthier diet quality.

## Introduction

As highlighted in the socioecological model of dietary behavior [1], physical food environments shape consumer choices by presenting barriers, opportunities, and varying levels of access and availability to meals and groceries. A spatial aspect of the physical food environment, is the distribution and diversity of food outlets within a specified geographical area, often defined as the ‘foodscape’, ‘community food environment’, or ‘physical food environment [2, 3]. The type of food outlet and how easy it is accessed are commonly employed measures for characterizing the foodscape, serving as indicators of the availability of specific food categories that impact the dietary behaviors and overall health of the exposed population [4–7]. Foodscapes that promote unbalanced diets towards the unhealthy are considered unfavorable at a public level.

Despite extensive research, evidence supporting expected associations between the foodscape and dietary outcomes remains inconsistent [8–10]. Fast-food outlets and supermarkets, the most studied outlet types, exhibit opposing influences on diets, with access to fast-food outlets being associated with poorer diet, with strongest support for such associations in the US [8–10]. Though sparsely studied, existing research on the Danish foodscape does appear to support the noted association. However, only convenience stores and fast-food outlets have been examined [11, 12]. Recent European studies mainly report no associations between different spatial measures of the food environment and dietary outcomes [13–15].

The foodscape of European countries differs from the US. Denmark, being a compact, highly urbanized nation, demonstrates a wide range of food outlets, facilitated by its concentrated urban population. Conversely, the US is characterized by lower population densities and sparsely populated regions with limited food outlet availability [16]. Moreover, as emphasized by the socioecological model [1], dietary choices are not solely determined by the physical structures. Cultural, economic, and personal factors also influence food decisions. Indeed, differences in associations across global regions, population groups, and contextual settings are suggested by several authors [8, 10, 17]. Even within Europe there is significant variability in these factors, especially between northern and southern countries [18].

Quantitative assessments of food availability commonly employ simple metrics like raw counts of a specific food outlet type or standardized counts adjusted for area or population (densities) [6]. One potential limitation of concentrating on such measures is that many studies tend to focus solely on exposure to one type of food outlet, disregarding the relative influence of various food outlets [5, 19]. However, while more complex measures such as proportions and ratios are intended to better capture the mix of different outlet types, they can potentially mask important variations in the actual differences in the foodscape across individuals or areas [5, 20]. In such cases, availability might be better reflected in simple measures. Thus, densities and proportions reflect different facets of the foodscape [7, 18–20]. Prior studies have demonstrated that measures considering the relative availability generally yield better model fit and more consistent directions of associations between the food environment and dietary patterns [21–23].

In Denmark, the prevalence of adults having very unhealthy dietary patterns has increased from 13% in 2013 to 17% in 2021, with marked geographical disparities [24, 25]. The Capital region demonstrates the lowest prevalence at 14.2%, whereas the North Denmark region exhibits the highest prevalence, at 20.9% [26]. Since studies on the Danish foodscape are limited, and the association with diet is expected to be context-dependent, this study aims to identify the most suitable statistical model for linking commonly used density and proportional food outlet availability measures to dietary quality within a Danish population characterized by varying prevalence rates of unhealthy dietary patterns. We discuss the impact of using food outlet density versus proportional availability measures by exploring their association with dietary quality.

## Methods

In this study, ChatGPT, a language model developed by OpenAI, was employed as a central tool for language editing.

### Study sample

Data from two Danish Regional Health Surveys conducted in 2017 were assessed [27, 28]. These surveys, which are part of the Danish National Health Surveys [29, 30] are cross-sectional questionnaires conducted every four years among citizens aged ≥ 16 years. Participants were randomly selected using the Danish Civil Registration System (CRS) from municipalities in the Capital and North Denmark regions. In the Capital region, the total 2017 sample included 55,185 individuals (response rate of 52.6%) [28], while the North Denmark Region included 22,583 individuals (response rate of 60.1%) [27]. All participants were geocoded based on exact home address obtained from the CRS and reference address data from the Danish Agency for Data Supply and Infrastructure. This allowed for the later construction of individual spatial measures and linking with individual level information on covariates. Further, the home address also facilitated the nesting of individuals within parishes for multilevel modeling purposes.

### Food outlet data and classification

Food outlet data from year 2017 in the National Food Safety and Hygiene Regulation Register (i.e., the Smiley Register) was provided upon request by the Ministry of Food, Agriculture and Fisheries of Denmark. Information on geocoordinates was applied to map all relevant food outlets within the study areas [31, 32]. Each food outlet was identified, located, and classified from predetermined criteria using a combination of (i) branch code, (ii) keywords/outlet name, (iii) Google Street View images and (iv) other information available online. We previously showed that this desk-based method had a positive predictive value (PPV) of 0.76, meaning that 76% of the food outlets identified in the Smiley Register were confirmed by data collected during street audits [33].Thus, the register does not reflect an exact copy of the foodscape but seems to represent the Danish foodscape reasonably, with a similar validity to many other secondary sources across countries [2]. The recorded locations of the food outlets were relatively close to the actual positions with a median ± IQR of 13.61 ± 14.23 m.

To ease comparability of the findings the analyses focus on four of the most commonly applied classifications in the spatial food environment literature (fast-food outlets, convenience stores, supermarkets and restaurants) (Table 1). The chosen outlet types and definitions are inspired by Wilkins *et. al* (2019a). Across food outlet types, the PPV’s for each of the food outlet classification were high (89–97%) for most of the food outlet types, except for greengrocers (44%) and restaurants (67%) [33]. Greengrocers and the miscellaneous classification, which included bakeries, butchers, and fishmongers, were counted in the total number of food outlets but not analyzed separately. Greengrocers were excluded from detailed analysis due to a low PPV but were included in the total count to maintain data representativeness. Although the miscellaneous classification had a high PPV, bakeries, butchers, and fishmongers are more relevant as separate classifications within the context of the study rather than as a combined category as identified by the desk-based method.

**Table 1:** Food outlet classifications and definitions in a Danish setting

Outlet type	Definitions
Fast-food	Major chain outlets (i.e. McDonalds, Sunset boulevard, Burger King, KFC, Subway, Dominos, Max burger); Non-chain traditional fast-food (e.g., small burger/kebab/pizza joints); Other fast-food takeaways (e.g., sushi, bagels, salad, street food, noodles) with no/limited seating and counter service/no waitress service
Convenience	Petrol station stores and small kiosks (i.e. Shell, Circle K, Uno X, OK plus, Q8, Kiosks); Convenience chains and corner stores with a limited selection of convenient items such as dairy products, frozen goods, fresh fruits and vegetables (i.e. 7-eleven Nærkøb, Letkøb, Elite købmand)
Supermarkets	Large chain supermarkets that often have long opening hours (e.g. 6am—11pm or 24h) and possibly a wide range of facilities such as clothes/homeware departments (i.e. Bilka, Kvickly, SuperBrugsen, Irma, Føtex, SuperBest, SuperSpar, EuroSpar, Løvbjerg, ABC lavpris, MENY); Supermarkets with shorter opening hours and a less extensive range of products and facilities (Discount supermarkets i.e. Fakta, Netto, Kiwi, Rema1000, Aldi)
Restaurants	‘Traditional restaurants’ serving primarily lunch/dinner meals and providing waited table service; Restaurants or cafés providing waited table service, serving readily prepared meals and snacks all day or buffet/brunch
Greengrocers	Market stalls or food outlets primarily selling fruits and vegetables and typically also long-life products but with a limited supply compared with supermarkets. Excluding farm shops
Miscellaneous	Bakeries, Butchers and Fish mongers

### Exposure – food outlet availability

Food outlet classifications were used to construct nine spatial measures reflecting the foodscape around home (Table 2). From raw food outlet counts, we constructed commonly applied density and proportion measures [34]. These comprised count of the four chosen food outlet types i) per unit area (density), and ii) relative to the total count (which additionally included greengrocers, as well as bakeries, butchers, and fish mongers). Further, we applied another common proportion measure: Count of fast-food outlets out of total count of fast-food outlets and restaurants [22].

**Table 2:** Spatial availability based on count of food outlets within 800m network buffers around home

	**Density**	**Proportion**
Fast-food outlets	Count per km^2^	Count per km^2^ out of all food outlets^a^ Count per km^2^ out of all eating out food outlets^b^
Convenience stores		
Supermarkets		
Restaurants		

^a^ All food outlets being the total count of fast-food outlets, supermarkets, convenience stores, restaurants, greengrocers and bakeries, butchers, and fishmongers. Applicable for fast-food outlets, restaurants, supermarkets and convenience stores

^b^ All eating out food outlets being the total count of fast-food outlets and restaurants. Applicable only for fast-food outlets, as it reflects their proportion relative to dining options

We used a commonly applied buffer size of 800 m around home as the geographical unit defining the neighborhood from which the exposures were constructed. We argue that a relatively small sized buffer reflects the immediate foodscape within a reasonable walking distance and increase the probability of its utilization by local residents. Street network data were obtained from ESRI’s ArcGIS StreetMap Premium 2022. Buffers were created in ArcGIS Pro 2.4.2 from the “Service areas” option, populating the facilities with the Universal Transverse Mercator (UTM) coordinates of participant addresses. The mode was set to “walking distance” with a cutoff at 0.8 km. The remaining travel settings was set as default. From the option “Summarize Within” we calculated the raw count of all food outlet types and the area of each network buffer. The options “Calculate field” and “Calculate geometry” were applied to compute the availability measures.

### Outcome – dietary quality score

The cross-sectional surveys assessed consumption frequencies of key components of a Danish diet. Participants reported how often they consumed 27 food items (including hot meals, side dishes, and vegetables) over the past week, with options of 0, 1–2, 3–4, or 5–7 times per week. For fruit intake, they indicated their daily consumption from eight categories, ranging from none to more than six pieces per day. To measure the overall quality of participants' diets, a validated dietary quality score (DQS) was utilized, based on consumption frequencies of fish, fruit, vegetables, and fat [35, 36]. For each food item the consumption frequency was scored on a scale of 0 to 2 points with higher values indicating a healthier dietary intake (Table 3). National recommendations, such as consuming a minimum of 600 g of fruit and vegetables daily and at least 300 g of fish weekly, formed the scoring system for fruit, vegetables, main course fish, and fish as cold cuts. The assignment of points for fats was influenced by considerations of their fatty acid composition and their determined nutritional and health attributes. The total fat score was derived from scores on the frequency of fat consumption, both on bread and for cooking, where up to 3 points could be assigned. Thus, overall, the DQS is intended as a comprehensive measure of overall dietary quality assessing dietary habits at a population level.

**Table 3:** Scoring system for the four food items included in the dietary quality score

	**Fruit**	**Vegetables**	**Fish**	**Fat**		
	Pieces/ 1 dL	Salad or cooked/raw vegetables or vegetable-vegetarian dishes	As main course or cold cuts	For cooking	On bread	Total fat
Points						
3	-	-	-	Predominantly cooking without fat or using olive oil	Mainly no spread	-
2	≥ 3 pieces/ day	≥ 1/ day or two types of vegetables ≥ 5 times/week	As main course ≥ 5–7 times/week or as cold cuts ≥ 3–4 times/week	Vegetable margarine, cooking/salad oil, corn/sunflower/grapeseed oil, or “mixed”	Minarine, vegetable margarine, or "mixed" spread	If 3 points in fat on bread, *and* fat for cooking
1	> 2 servings/week but < 1/day or two types of vegetables ≥ 5 times/week	> 2 servings/week but < 1/day or two types of vegetables ≥ 5 times/week	≥ 1/week but < 5–7 times/week as main course or < 3–4 times/week as cold cuts	Predominantly using frying margarine/ butter/margarine spread/fat/palm oil	Mainly butter, margarine spread, or fat	If 3–5 points in total
0	≤ 2 pieces/ week	≤ 2 servings/week	No intake in the last week			If 1 point in fat on bread, and fat for cooking

Overall scores of the DQS ranged from 0 to 8 points designed to distinguish between two smaller extreme groups (very unhealthy/healthy) and a larger intermediate group (3–5 points), thereby capturing a broad range of dietary patterns. In this study, the DQS was treated as a continuous variable with higher scores indicating healthier dietary quality. The DQS has been validated by Rostgaard-Hansen et al*.* (2023) sing a 376-item food frequency questionnaire (FFQ) and concentration biomarkers in a sample of 450 participants (75% response rate). The results showed that a higher DQS was significantly associated with better overall dietary quality. This was associated with an increased consumption of fruits, vegetables, fish, fiber, vitamins, and minerals, along with more favorable health markers such as lower levels of LDL cholesterol, Hs-CRP, waist circumference, visceral fat, total fat mass, and total fat percentage, as well as higher HDL cholesterol levels (*P* < 0.05). The spearman’s correlations coefficients between the intake of fish, fruit, and vegetables in the 376-item FFQ and the 23-item FFQ were 0.63, 0.61 and 0.31 respectively.

### Covariates

Each spatial measure was modelled separately across food outlets type, controlling for the following individual covariates: age (continuous), gender (male;female), ethnicity (danish; western; other), highest completed education (student; primary school; upper secondary school; vocational education; academy or bachelor degree; master or PhD degree), yearly income (continuous) and labor market affiliation (employed; in education; unemployed; long-term illness, vocational rehabilitation or social welfare benefit; early retirement; retired). Age, gender and ethnicity were drawn from the CRS [37]. Ethnicity classifications were developed by Statistics Denmark based on information regarding the birthplaces and citizenship of the parents [38]. Thus, a person is considered Danish if at least one parent is both a Danish citizen and born in Denmark. Information on education, income and labor market affiliation were drawn from other population registries [39–41].

At the municipal level, an aggregated covariate denoting urbanicity was applied. Urbanicity classifications were developed by Statistics Denmark in 2018, categorizing municipalities into five groups (capital; metropolitan; provincial; commuter; rural) based on job availability and the population of the largest city within the municipality in 2017. Job availability was computed by Statistics Denmark, weighing distances to workplaces at the parish level and then aggregating them to the municipality level. This process assigned higher job availability to municipalities closer to a greater number of workplaces [42]. As such, the Capital and metropolitan municipalities both housed ≥ 100,000 residents but had job availabilities of ≥ 200,000 and < 200,000, respectively. Provincial municipalities also had a job availability of ≥ 200,000 but were home to between 30,000 and 100,000 residents. Commuter and rural municipalities encompassed < 30,000 residents but had job availabilities of ≥ 40,000 and < 40,000, respectively.

Covariates were chosen based on previous research that has demonstrated the impact of age, gender, educational level, ethnicity, and socioeconomic background on dietary habits [25, 43, 44]. Moreover, there are significant disparities in food outlet availability for individuals residing in capital, urban, and rural areas, especially with smaller buffer sizes [23]. The chosen covariates are also commonly controlled for in similar studies [45].

### Statistical analyses

Analyses were conducted using SAS Enterprise Guide 7.1 with survey procedures. Participant characteristics were summarized and chi-square tests were used for analyzing group differences for categorical variables, while ANOVA was used to assess differences in continuous variables among the participants. The DQS was treated as continuous and checked for normality via histograms and residual plots. Separate multilevel linear regression models were used for each of the four food outlet types. These models account for the fact that individuals residing within the same area tend to be more alike given that they are exposed to the same factors (Voigtländer, et al., 2013). A random intercept model (proc GLIMMIX) was employed, featuring two levels: health survey participants (level 1) nested within parishes (level 2). Parish administrative boundaries determined the area-level random effects, while individual and area characteristics were fixed effects. To ensure complete nesting, parishes that were not fully nested within a single municipality (< 1%) were manually assigned based on official sources on parish [46]. Initial multilevel regressions were performed to explore the association between each of the nine availability measures and DQS. Linear and natural cubic splines were modelled (EFFECT statement) with knot placement according to Frank Harrell’s recommended approach, i.e., with knots fairly close to the extreme values and then evenly distributed internal knots. Linear spline knots were adjusted based on residual plots and Akaike Information Criterion (AIC) values for optimal model fit. Models including significant cubic or linear splines (*p* < 0.05) with lowest AIC were considered to have a better fıt. Finally, linear regression models guided by AIC were compared against significant spline models.

Proportion measures did not necessitate spline modeling, whereas density measures were modeled using linear splines with different knots based on the food outlet type. For instance, fast-food density utilized a linear spline model representing a piecewise linear interpolation with one knot at 5 counts per km^2^, consequently producing two parameter estimates with accommodating p-values and 95% confidence intervals. Similarly, convenience and supermarket density were both included in the models with one knot at 3 counts per km^2^. Restaurant density was included in the model with two knots at 3 counts per km^2^ and 31 counts per km^2^. A significant proportion of the observations contained zero values, which was expected given the small buffer sizes used in the analysis. Importantly, model diagnostics confirmed that the overall distribution of the data and the residuals adhered to the assumptions of normality and linearity, suggesting that the inclusion of zero values did not adversely affect the model's performance. Therefore, these zero values were retained in the dataset.

We employed a complete-case analysis approach, excluding any cases with missing data on the variables included in the model. We excluded 5,911 participants due to missing information on either: food outlet availability (*N* = 29), DQS (*N* = 3,646), parish (*N* = 58), labor market affiliation (*N* = 1638), yearly income (*N* = 12), and highest completed education (*N* = 926). Consequently, the final analysis was conducted on a subset of the original data, where all relevant variables were fully observed (*N* = 71,857). Each exposure was analyzed using three models: Model 1 included DQS and food outlet availability; Model 2 added age, gender, ethnicity, labor market affiliation, income, and educational level; Model 3 was fully adjusted, incorporating Model 2 variables and urbanicity. Findings from fully adjusted models were applied to report associations between availability measures and the DQS. Beta coefficients represented the DQS difference for each increase in exposure (percentage or count per km^2^) for either a simple regression line or linear splines up to first knot, between knots and above the largest knot. For instance, associations with fast-food density, are presented as beta coefficients representing the difference in DQS for each count of fast-food outlets per km^2^ up to 5 counts per km^2^ and above 5 counts per km^2^. We do not account for multiple comparisons in our analyses by adjusting with e.g., a Bonferroni correction in the models. Instead, we illustrate unadjusted findings in the tables with accompanying p-values (P), but discuss findings from a threshold for statistical significance based on Bonferroni adjusted *p*-values P^’^ = k*P < 0.05, where k is the number of tests [47]. In total nine tests were conducted, one for each exposure. i.e., the threshold used for interpretation is *P*^’^ < 0.05/9 = 0.006.

Associations were compared using parameter estimates, p-values, AIC values, and Akaike weights, presented for the fully adjusted model 3. Akaike weights were calculated according to Wagenmakers and Farrell (2004) [48] indicating the relative performance of fitting density versus proportions across each food outlet type, from evidence ratios. Akaike weights can be interpreted as the probability that a model is the best model given the data and the candidate model. The strength of evidence in favor of one model over another is obtained by dividing their Akaike weights (i.e., the evidence ratio).

## Results

Our study sample included 77,768 participants aged 16 and older. The final sample for analysis comprised 71,857 participants with a mean age of 51.7 years and a mean income of 366,840 Danish Krones (DKR) equivalent to approximately 49,200 euro/54,200 US dollars. Significant differences in the DQS were observed across groups for all covariates. A higher mean DQS was observed among women, individuals of Western origin, and those with a higher level of education (Table 4). Employed individuals and those with a bachelor’s, master’s, or PhD degree also had a higher mean DQS. At the area level, the highest mean DQS was found among residents in the Capital and provincial areas.

**Table 4:** Mean dietary quality score across characteristics of the 71,840 study participants from the two Danish Regional Health Surveys conducted in the Capital and North Denmark Region, 2017. Dietary quality score ranges from 0–8 points where higher scores represent healthier eating habits. Data not weighed to account for the difference in non-response

	**Mean**	**SD**	**Number**
**Dietary quality score**	4.21	1.54	71,840
Gender*			
Women	4.42	1.48	39,292
Men	3.95	1.57	32,548
Ethnicity*			
Danish	4.18	1.54	65,686
Western background	4.49	1.39	2,513
Other	4.48	1.45	3,641
Labor market affiliation*			
Employed	4.28	1.50	41,155
In education	4.11	1.53	6,462
Unemployed	3.99	1.57	678
Long-term illness, vocational rehabilitation, or social welfare benefit	3.84	1.66	2,307
Early retirement	3.78	1.73	1,832
Retired	4.18	1.57	19,406
Highest completed educational level*			
Student	4.08	1.53	7,171
Primary school	3.82	1.64	12,110
Upper secondary education	4.26	1.47	3,624
Vocational education	3.97	1.56	23,057
Academy or bachelor degree	4.56	1.41	16,760
Master or PhD degree	4.75	1.30	9,118
Municipality urbanicity*			
Capital	4.32	1.49	41,201
Metropolitan	3.99	1.54	3,525
Provincial	4.33	1.50	2,561
Commuter	4.22	1.57	7,909
Rural	3.95	1.59	16,644

^*^ Chi^2^ test *p* < 0.0001

Within the study area we identified a total of 7,262 food outlets. The distributions across outlet type were as follows: 1,937 fast-food outlets, 834 convenience stores, 874 supermarkets, 2,695 restaurants, 203 greengrocers and 719 food outlets in the miscellaneous category. In Table 5 the mean or median are given across classifications and for the total count. One third of the participants had more than 15 food outlets within their neighborhood, however the discrete nature of the food outlet data gave rise to zero-values in the densities. For instance, half of the participants had a convenience store density of zero. Two thirds had a fast-food density of 4.64 or less in their residential food environment. The corresponding densities for convenience stores, supermarkets and restaurants were 2.12, 2.52 and 4.11, respectively. The highest maximum densities were found for fast-food outlets and restaurants with counts of 70.21 and 174.37, respectively, per km^2^. For the relative measures there were neighborhoods with zero counts of the chosen food outlet and zero counts of other outlets. The mean proportion out of all food outlets that were fast-food outlets was 22%. The corresponding proportions for convenience stores, supermarkets and restaurants were 11, 14 and 19%, respectively. A mean proportion of 37% out of all “eating out” options were fast-food.

**Table 5:** Mean or median availability within individual 800m network buffers around home. *N* = 71,857

Food outlet availability							
		Median	Mean	Variance	IQR	Min	Max
**Fast-food**	Density ^a^	1.25	-	-	4.64	0	70.21
	Proportion ^b^	-	0.22	0.05	-	0	1
	Proportion ^c^	-	0.37	0.13	-	0	1
**Convenience stores**	Density ^a^	0.00	-	-	2.12	0	23.18
	Proportion ^b^	-	0.11	0.03	-	0	1
**Supermarkets**	Density ^a^	0.92	-	-	2.54	0	17.20
	Proportion ^b^	-	0.14	0.04	-	0	1
**Restaurants**	Density ^a^	0.93	-		4.11	0	174.38
	Proportion ^b^	-	0.19	0.05	-	0	1
**All food outlets**	Total count ^d^	4.00	-	-	15	0	429

*IQR* Inter quartile range

^a^ Counts per km^2^

^b^ Out of all food outlets including fast-food, convenience stores supermarkets, restaurants, and specialized food outlets

^c^ out of all “eating out” options including fast-food and restaurants

^d^ Raw counts of fast-food, convenience stores supermarkets, restaurants, and specialized food outlets

Model fit values and associations between availability measures and the DQS are found in Table 6. Regardless of outlet type all densities were significantly negatively associated with the DQS below the first knot and positively associated with the DQS above the first knot, in the fully adjusted models. For instance, each increase in fast-food count per km^2^ up to 5 was associated with a decrease in the DQS by 0.017 points (i.e., 0.2%) towards unhealthier eating habits. Fast-food densities above 5 were associated with an increase in the DQS towards healthier eating habits by 0.023 points (i.e., 0.3%) for each count per km^2^. Same patterns were seen for density measures of convenience stores, supermarkets, and restaurants. However, for restaurant densities above 31, the direction of association reversed and became significantly negative again. When correcting for multiple comparisons the only significant effect size (*p* < 0.006) among the proportion measures was the proportion of fast-food out of all “eating out” options. This measure was associated with a decrease in DQS by 0.058 (i.e., 0.7%) for each percent increase in proportion.

**Table 6:** The association between availability and the dietary quality score (DQS) across food outlet types displayed with beta coefficients, 95% CI, p-value and further Akaike Information Criterion (AIC), Akaike weights and Evidence ratio of Akaike weights in model 3. DQS ranges from 0–8 where higher scores represent healthier eating habits. Model 1 include DQS and availability; Model 2 add age, gender, ethnicity, labor market affiliation, income, and educational level; Model 3 is fully adjusted, incorporating Model 2 variables and urbanicity. Parameter estimates in the table are not corrected for multiple comparisons. Data not weighed to account for the difference in non-response. *N* = 71,851

**Availability**^**1**^	**Model 1**	**Model 2**	**Model 3**	
**Density**^**2**^	**Estimate (95% CI) [*p*-value]**	**Estimate (95% CI) [*p*-value]**	**Estimate (95% CI) [*p*-value]**	**AIC (Model 3) (Akaike weights**^**a**^**) [Evidence ratio**^**b,c**^**]**
Fast-food				
0–5	**-0.010**	**-0.007**	**-0.017**	259,464
	(-0.018;-0.002)	(-0.0147;0.0003)	(-0.025;-0.009)	(0.9817)
	[0.0167]	[0.0603]	[< 0.0001]	[2980]
> 5	**0.018**	**0.014**	**0.023**	[55]^c^
	0.008;0.027	(0.006;0.023)	(0.015;0.032)	
	[0.0003]	[0.0014]	[< 0.0001]	
Convenience				
0–3	**-0.022**	**-0.013**	**-0.023**	259,467
	(-0.037;-0.008)	(-0.26;0.0003)	(-0.042;0.023)	(0.9817)
	[0.0027]	[0.06]	[< 0.0001]	[55]
> 3	**0.045**	**0.034**	**0.045**	
	(0.025;0.064)	(0.012:0.051)	(0.028;0.063)	
	[< 0.0001]	[0.0002]	[< 0.0001]	
Supermarkets				
0–3	**-0.021**	**-0.011**	**-0.022**	259,479
	(-0.033;-0.008)	(-0.023;0.0002)	(-0.034;-0.010)	(0.1192)
	[0.001]	[0.056]	[0.0002]	[0.14]
> 3	**0.037**	**0.030**	**0.036**	
	(0.0154;0.060)	(0.009;0.050)	(0.016;0.056)	
	[0.0009]	[0.0045]	[0.0004]	
Restaurants				
0–3	**-0.022**	**-0.016**	**-0.025**	259,471
	(-0.033;-0.009)	(-0.028;-0.005)	(-0.036:-0–013)	(0.9933)
	[0.0005]	[0.0041]	[< 0.0001]	[148]
> 3–31	**0.031**	**0.024**	**0.032**	
	(0.017;0.0443)	(0.012;0.034)	(0.019;0.044)	
	[< 0.0001]	[0.0002]	[0.002]	
> 31	**-0.007**	**-0.007**	**-0.006**	
	(-0.012;-0.003)	(-0.011;-0.003)	(-0.009;-0.002)	
	[0.0012]	[0.0004]	[0.002]	
**Proportion**^3,4^				
Fast-food^3^	**0.032**	**0.023**	**-0.038**	259,480
	(-0.027;0.0091)	(-0.032;0.078)	(-0.093;0.018)	(0.0003)
	[0.29]	[0.41]	[0.18]	
Convenience				259,475
	**-0.050**	**-0.038**	**-0.080**	(0.018)
	(-0.119;0.018)	(-0.103;0.027)	(-0.145;-0.015)	
	[0.15]	[0.26]	[0.016]	
Supermarkets				259,475
	**-0.076**	**-0.050**	**-0.073**	(0.881
	(-0.135;-0.018)	(-0.107;0.004)	(-0.128;-0.017)	
	[0.01]	[0.07]	[0.01]	
Restaurants				259,481
	**0.034**	**0.035**	**0.016**	(0.0067)
	(-0.020;0.088)	(-0.017;0.086)	(-0.034;0.067)	
	[0.22]	[0.17]	[0.53]	
Fast-food^4^				259,472
	**-0.014**	**-0.013**	**-0.058**	(0.018)
	(-0.052;0.023)	(-0.048;0.022)	(-0.093;-0.022)	
	[0.46]	[0.47]	[0.0017]	

^1^ Within a residential 800m network buffer

^2^ Count per km^2^

^3^ Proportion out of all food outlets (fast-food, convenience stores supermarkets, restaurants, and specialized food outlets)

^4^ Proportion of all “eating out” opportunities (fast-food and restaurants)

^a^Akaike weights determined for models with availability measures (density vs. proportion) within each outlet type

^b^ Akaike weights from model with food outlet density divided by Akaike weights from model with food outlet proportions for each outlet type

^c^ Akaike weights from model with fast-food density /fast-food proportion of all “eating out” opportunities

Model fit for models including densities as exposure were generally preferred over models including proportions. For instance, a raw AIC value of 259,467 from the model including convenience density is smaller than an AIC value of 259,475 from the model including the proportion of convenience stores. The corresponding Akaike weights and evidence ratio support that the best fitting model is the one including the density measure which is 55 times more likely to be the best model relative to the model fitting the corresponding proportion measure.

## Discussion

In a large population with detailed information on sociodemographic characteristics we assessed the association between a crude measure of dietary quality and the foodscape, quantified from both density and proportion measures for various types of food outlets. We identified significant associations between the DQS and food outlet density across various outlet types. Notably, these were all negatively associated with the higher dietary quality according to the DQS until reaching densities of 3–5 (count/km^2^), at which point the direction of association shifted towards a healthier DQS. When considering the presence of competing food outlets, we observed that a higher proportion of fast-food outlets among all “eating out options” was significantly associated with a lower DQS as the proportion increased. However, other proportion measures did not show significant associations with the DQS after accounting for multiple testing.

### Interpretation of findings

Generally, food outlet densities yielded more consistent direction of associations, significant but modest effect sizes and better model fit. This highlights the importance of the chosen spatial measure in quantifying the foodscape. The finding was interesting, as one might expect that proportions, rather than densities, to better describe the association with the overall dietary pattern as these theoretically get closer in representing the foodscape. The observed change in association suggests a threshold effect, where increased food outlet density is associated with healthier dietary patterns, indicating that abundant food options may promote healthier choices. However, distinguishing the impact of different food outlet types, particularly in urban areas with high food availability, is challenging. The increased effect sizes and significance from model 2 to model 3 highlight the influence of urban structures. Additionally, residual confounding may be present if we have not adequately accounted for factors affecting dietary choices in areas with high food outlet density. That the increasing proportion of fast-food outlets relative to restaurants was associated with a decrease in dietary quality, could suggest a substitution effect where greater availability of fast-food leads people to choose these unhealthier options, resulting in a lower DQS.

The consistent pattern in the direction across all types of food outlets was unexpected and challenges the conventional belief that supermarkets offer mostly healthy options. In fact, this has recently been contradicted in a Dutch study where 80% of both the food assortment and the in-store products and prices featuring in supermarkets’ promotions were not supportive of a healthy diet [49]. The unexpected direction of associations may be attributed to factors such as socioeconomic status, urbanicity, and population density, which could influence food outlet availability, in-store offerings, and dietary quality [8, 50–53]. For instance, spatial disparities in access to both fast-food and supermarkets. The strongest and most consistent findings relate to fast-food outlets, particularly in the U.S., Canada, the UK, and Australia, where lower socioeconomic areas often have greater access, leading to the phenomenon of food swamps [8, 50, 54]. In contrast, in the U.S., a consistent association is observed between lower socioeconomic areas and reduced access to supermarkets and grocery stores, described as food deserts [8, 55]. However, studies in other developed countries show mixed results [8, 52, 56–58]. In Copenhagen, low- and mid-low-income neighborhoods experienced poorer availability of fast-food outlets, but not supermarkets [58]. Other data (unpublished) from the Capital region of Denmark indicate that disadvantaged areas are more likely to have fast-food outlets, supermarkets, and convenience stores within an 800m buffer, compared to more advantaged areas, but only when accounting for urbanicity [53]. Further, while the chosen food outlet classifications in the present study closely align with previous research [34], the definition of fast-food outlets extends beyond high-salt, energy-dense options to include healthier choices like sushi and salads. Similarly, the convenience store category encompasses not only corner stores and gas stations but also small-scale supermarkets. Thus, considering food outlets as simply healthy or unhealthy may be inadequate due to the diversity of their offerings. It is recommended to validate this binary categorization by assessing the actual products using tools like the Nutrition Environment Measures Survey (NEMS) [59, 60]. Alternatively, consider using an aggregate measure of food outlet quality, such as the Food Environment Healthiness Index developed by Chan et al. [13], which classifies outlets based on the nutritional quality of their offerings using the Delphi method.

The similarity in associations across food outlet types may also be linked to the spatial access measure used. Findings from a review by Bivoltsis et al*.,* (2018) suggests that proximity to a supermarket (accessibility) may be more important than the count (availability) in influencing fruit and vegetable intake, whereas the count of fast-food and convenience stores may more strongly be associated with unhealthy food intake.

### Comparison with previous studies

Previous research have demonstrated that more complex measures of food outlet availability offer better model fit and more consistent food environment-diet associations [21–23], though this pattern is not consistent across all studies [19, 61]. In a study of the foodscape and diet in urban regions across five European countries, Pinho et al*.* [19] found no significant associations with either density measures or more complex metrics like the proportion measures applied in the present study. Interestingly, the direction of association between healthy and unhealthy food outlets was similar. However, this direction varied depending on whether densities or complex metrics were used to quantify the foodscape, with densities associated with less healthy dietary patterns, and more complex metrics associated with healthier patterns. In the present study, the direction of associations was consistent and significant across all density measures, but the most substantial effect was observed for the one significant proportion measure. It is plausible these findings are rather sensitive to methodological choices such as statistical models and underlying assumptions [62]. While our method addresses complexities by allowing for multilevel influence from both individual and parish in a nested design, we acknowledge that applying alternative models, such as Generalized Estimating Equations (GEEs) and might yield different results. Generally, our current understanding of how the foodscape influences diet and diet-related outcomes is limited by methodological differences across studies, which limit comparability [4, 7, 19, 22, 23, 63, 64]. For instance, quantifying the foodscape involves considerations regarding type of measure (e.g., accessibility, availability, or both), whether it should be simple (number or densities) or more complex (proportions, gravity measures), individual or at an area level. Defining the area from which the access metric is derived (e.g., around home, school, travel path) and setting geographical boundaries (e.g., administrative, buffer size, or activity space) adds to the complexity. However, as suggested, differences in cultural, economic, and social norms may also be reflected in the different associations found across countries. A recent Dutch study [13] and UK study [14] that methodologically resemble our research conclude no association between the foodscape and diet [13, 14] while a recent French study [15] find only simple availability measures of some food outlet types to be significantly associated with quality of food purchases for home consumption. More specifically, significant associations were observed with the number and presence of markets, which were associated with poorer nutritional quality of food purchases. Conversely, greengrocers were associated with better nutritional quality. Supposedly, France and the Netherlands may to some extend resemble Denmark with regards to socioeconomic structures and urban configurations, however cultural and social norms differ across population groups. Hence, the differing findings support the relevance of considering context-specific associations.

### Strengths and limitations

We employed a comprehensive approach by considering various and commonly applied types of food outlets, validated food outlet data, and a validated dietary quality score to assess associations between the foodscape and diet quality in Denmark. We utilized large sample sizes and employed multilevel models to account for correlations in both dietary habits and food outlet availability among individuals living in the same area. Our analysis encompassed large and diverse geographical areas, which is necessary for capturing variations in foodscapes. Additionally, the spatial scale of the foodscape was determined from individual fixed points, enhancing the accuracy of exposure assessment to specific food outlets in comparison to utilizing area fixed points [65]. Applying network buffers to shape the boundaries of the foodscape accommodates the consideration of physical barriers that could influence the selection of a food outlet. However, we acknowledge that a buffer is limited by its binary representation of food outlets as it is either included or excluded, assuming that the impact of a food outlet is uniform within the buffer and that it abruptly stops beyond the border. The examined foodscape is further limited by being quantified only from residential areas which may have led to an incomplete representation of an individual’s true exposure to food environments [65, 66]. Individuals do not necessarily shop at food outlets that are closest to home [67–69]. Instead, they may consider other factors such as price, product selection, quality, convenience, or personal preferences when deciding where to shop. Other relevant areas to consider includes areas around work, school, and recreational areas. Recently, Marwa et al*.,* (2021) demonstrated in a small study sample (*N* = 65) that the size of activity space was directly related to the distance from home to workplace. Defining the relevant foodscape using activity space notably outperformed conventional buffers like those applied in our study. Further, buffers encompassing both home and workplaces aligned better with activity space than those focusing on one location. While incorporating larger buffer sizes in the current study was an option, this was not directly related to the main aim. The chosen 800m buffer, is approximately equivalent to a 10-min walk [70], suitable for densely populated or less mobile populations [71], is commonly used in food environment studies, and a typical walking distance for food purchases in Denmark [72]. However, we acknowledge that this buffer size may not be appropriate for all food outlet types, especially in areas with lower population densities [73]. Thus, while efforts were made to align with commonly used methods in the literature, the classifications and quantification of the foodscape remain subjective and do not fully capture a unique Danish context.

The study did not consider the influence of food delivery services, which are increasingly popular in Denmark [74] and pose a challenge to traditional methods of studying the foodscape's impact on diet. Additionally, there are limitations to the DQS because it relies on recalled self-reported dietary habits, which could result in over- or underreporting and introduce bias related to participant characteristics [30, 75]. As previously stated, Rostgaard-Hansen et al*.* (2023) conducted a validation of the DQS in 2016, affirming its reliability. Notably, the DQS did not exhibit strong correlations with specific individual food items when compared to the reference FFQ. Thus, it is possible that the DQS may not effectively capture all noteworthy associations in our study. Finally, findings are also limited by the cross-sectional study design making it difficult to determine whether foodscapes influence diet or if dietary choices prompt individuals to engage in foodscape self-selection [76]. Additionally, food environments are dynamic and change over time [77, 78]. To better establish the sequence between foodscape exposure and dietary behaviors, longitudinal designs are essential. For instance, natural experiments assessing changes in food access due to build environment interventions, offer strong causal inference [9].

### Implications for future research

In areas where the foodscape is sparsely studied, such as Denmark, smaller-scale studies could benefit from examining individuals' daily paths, including their activity spaces, or exploring the multiple foodscapes they encounter throughout the day. This approach would provide a more comprehensive understanding of the relevant foodscape for individuals, including its size, boundaries, and other characteristics [17, 66]. In larger populations studies, applying a fixed location to define the local foodscape represents a feasible approach for quantifying exposure-outcome associations. However, it would be relevant to learn more about the effect of various buffer sizes that incorporate home and workplace locations, while also considering participants’ commuting patterns. Further, it is also relevant to explore other spatial indicators such proximity measures [6, 65] or kernel density [4, 5, 34]. As a final note we would like to emphasize, that while studying the spatial aspects of the physical food environment is essential for understanding the complex associations between the food environment and dietary patterns, other non-spatial factors, as emphasized by socio-ecological models, also play a significant role [1, 79]. Thus, the impact of foodscapes might be relatively small when compared to e.g., in-store attributes or government regulations that can impact food outlet and food availability.

## Conclusion

Generally, food outlet densities rather than proportional measures of the Danish foodscape performed better in linear regression multilevel models, showing better model fit, significant and consistent direction of associations with the DQS of adults. The cross-sectional associations found between food outlet densities and the DQS were complex and may be best described as negative up to a certain threshold around 3–5 counts per km^2^ beyond which the association with the DQS became positive (moved in a healthier direction). Contrary to expectations, the direction of associations remained fairly consistent across different food outlet types. This could suggest that the classifications fail to accurately depict intended food outlets or that the conventional categorization of food outlet types as healthy or unhealthy might be too simplistic.

## Data Availability

Data sharing is not applicable to this article due to compliance with the General Data Protection Regulation (GDPR) privacy regulations.

## Abbreviations

AIC: Akaike Information Criterion
ANOVA: Analysis of Variance
CRS: The Danish Civil Registration System
DKR: Danish Krone
GEEs: Generalized Estimating Equations
IQR: Inter Quartile Range
NEMS: Nutrition Environment Measures Survey
PPV: Positive Predictive Value
SES: Socioeconomic status
UTM: Universal Transverse Mercator
